# Machine learning models in clinical practice for the prediction of postoperative complications after major abdominal surgery

**DOI:** 10.1007/s00595-023-02662-4

**Published:** 2023-02-25

**Authors:** Wessel T. Stam, Erik W. Ingwersen, Mahsoem Ali, Jorik T. Spijkerman, Geert Kazemier, Emma R. J. Bruns, Freek Daams

**Affiliations:** 1 Department of Surgery, Amsterdam UMC Location Vrije Universiteit Amsterdam, De Boelelaan 1117, 1081 HV Amsterdam, The Netherlands; 2 Cancer Center Amsterdam, Cancer Treatment and Quality of Life, Amsterdam, The Netherlands; 3 AGEM Amsterdam Gastroenterology, Endocrinology and Metabolism, Amsterdam, The Netherlands; 4 Independent Consultant in Computational Intelligence, Amsterdam, The Netherlands

**Keywords:** Machine learning, Prediction, Postoperative complications

## Abstract

Complications after surgery have a major impact on short- and long-term outcomes, and decades of technological advancement have not yet led to the eradication of their risk. The accurate prediction of complications, recently enhanced by the development of machine learning algorithms, has the potential to completely reshape surgical patient management. In this paper, we reflect on multiple issues facing the implementation of machine learning, from the development to the actual implementation of machine learning models in daily clinical practice, providing suggestions on the use of machine learning models for predicting postoperative complications after major abdominal surgery.

## Introduction

Surgical patients are inevitably at risk of suffering postoperative complications, despite decades of scientific and technological advancement. The accurate prediction of individual outcomes has the potential to completely reshape the future of postoperative management. Such prediction would enable shared clinical decision-making and individual perioperative care and postoperative management.

In the last few years, a number of prediction models have been developed using machine learning (ML) models. These models offer the opportunity to develop a more individualized approach, allowing for data-driven individualized medicine [1–3]. However, clinical implementation and acceptance are cumbersome, as it is often hampered by non-compliance with necessary guidelines.

To achieve the transparent, safe, and applicable implementation of ML models in the prediction of postoperative outcomes, we propose uniform selection, training, and guideline compliance.

## Barriers and solutions

There is a large gap between promising and comprehensive research on the potential utility of artificial intelligence (AI) in the field of medicine and its actual implementation in daily clinical practice [4]. Several authors have tried to use ML models to optimize postoperative management by predicting postoperative complications. Unfortunately, many conclude that the implementation of these models is far from being clinically viable, even though most ML models achieve reasonable performance [5–15]. Cao et al. [5], Weller et al. [12], and Van den Bosch et al. [15] concluded that no practical implementation could be achieved for ML models due to the predictive value being too low to clinically implement. Grass et al. predicted surgical site infection in patients after colorectal surgery and initially found that ML outperformed conventional logistic regression. However, after external validation the practical applicability dropped due to low predictive performance [7].

Table 1 summarizes the outcomes of the largest, most recent articles on postoperative complication prediction with ML in major abdominal surgery. The shift to clinical implementation will depend on five main improvement categories: technology, policy, medical and economic impact, transparency, and reporting [4, 16, 17].

**Table 1:** Description of recent large-scale studies on ML and complication predictions as well as key findings

First author (year)	Study design	Data source	Population	Prediction	Methods	Outcome
Cao (2019)	Retrospective	National database	*N*: 44,061 Surgical specialty: bariatric surgery Number of patients with complications: 1408 (3.2%)	30-day severe postoperative complications	Comparison of 29 ML models Internal and external validation Overfitting prevention	No achievement of practical applicability due to low performance
Chen (2018)	Retrospective	Institutional database	*N*: 13,399 Surgical specialty: colorectal surgery Number of patients with complications: 1680 (12.5%)	7-day postoperative bleeding	Comparison of an ML model to conventional LR Internal validation, no external validation Overfitting prevention	ML models outperformed conventional LR models. They potentially allow for targeted surveillance
Grass (2020)	Retrospective	National database	*N*: 2376 Surgical specialty: colorectal surgery Number of patients with complications: 108 (4.6%)	Surgical site infection	Comparison of an ML model to conventional LR Internal validation, no external validation	ML models outperformed conventional LR models. However external validation showed low performance. Institutional data were advised
Han (2020)	Retrospective	Institutional database	*N*: 1769 Surgical specialty: pancreaticoduodenectomy Number of patients with complications: 221 (12.5%)	Postoperative pancreatic fistula	Two ML models tested Internal validation, no external validation	ML models outperformed conventional LR models
Merath (2020)	Retrospective	National database	*N*: 15,657 Surgical specialty: liver (685 [4.4%]), pancreatic (6012 [38.4%]) and colorectal (8960 [57.2%]) surgery Number of patients with complications: 6073 (38.8%)	30-day postoperative complications	Comparison of an ML model to conventional risk scores Internal validation, no external validation	ML models outperformed conventional LR models
Nudel (2021)	Retrospective	National database	*N*: 436,807 Surgical specialty: bariatric surgery Number of patients with complications: 3,068 (0.70%) developed anastomotic leakage, 2012 (0.46%) developed venous thromboembolism	30-day postoperative anastomotic leakage and venous thromboembolism	Comparison of two ML models to conventional LR Internal and external Overfitting prevention	ML models outperformed conventional LR models regarding anastomotic leakage LR models outperformed ML models regarding venous thromboembolism
Pera (2022)	Retrospective	National database	*N*: 3182 Surgical specialty: gastric surgery Number of patients with complications: 178 (5.6%)	90-day postoperative mortality	Comparison of three ML models to conventional LR Internal validation, no external validation Overfitting prevention	Good ML model performance, but due to no external validation and an imbalanced dataset. outcomes may not be extrapolatable
Shi (2012)	Retrospective	National database	*N*: 22,926 Surgical specialty: liver Number of patients with complications: 619 (2.7%)	In-hospital mortality	Comparison of two ML models to conventional LR Internal and external validation	ML models outperformed conventional LR models
Van den Bosch (2022)	Retrospective	National database	*N*: 62,501 Surgical specialty: colorectal surgery Number of patients with complications: 1693 (2.7%)	30-day postoperative mortality	Comparison of four ML models Internal validation, no external validation Overfitting prevention	ML models outperformed conventional LR models. However, there was no achievement of practical applicability due to low performance
Weller (2018)	Retrospective	Institutional database	*N*: 9598 Surgical specialty: colorectal surgery	Postoperative complications	Comparison of four ML models Internal and external validation	There was no achievement of practical applicability due to low performance
Wise (2019)	Retrospective	National database	*N*: 101,721 Surgical specialty: bariatric surgery Number of patients with complications: 3,853 (3.8%)	30-day postoperative morbidity and mortality	Comparison of an ML model to conventional LR Internal validation, no external validation	LR models outperformed ML models

*N* number, *ML* machine learning, *LR* logistic regression

### Selection of a model

Model selection in conventional statistics is well defined due to strict requirements, as opposed to ML models, in which it typically depends on several factors. Even experienced data scientists have difficulty selecting an optimal model [18].

Model selection in ML, which involves determining the highest overoptimism-corrected performance in the metric and output of choice, is performed post hoc by exploring different models. Factors of consideration are the quality and size of the data, questions that must be answered, time available for running the model, and type of desired output. Depending on the hypothesis, different metrics can be used to measure this performance. For example, in the prediction of events with a relatively low incidence (e.g. anastomotic leakages after hemicolectomy), imbalanced data may occur. In such cases, using a single metric to score outcome such as accuracy is less effective, therefore, precision, recall, and the *f*1-score are more explanatory. Although it is advisable to use multiple metrics to measure performance more broadly, most studies published thus far on the use of AI for predicting complications did not adhere to this principle. The majority of such studies focused only on sensitivity, specificity, and the area under the receiver operating characteristic curve (AUROC) [19, 20].

The complexity of ML models may lead to challenges in understanding the involved mechanisms [21]. Many argue that it is highly inadvisable to rely on outcomes of ‘black-box’ systems in the decision-making process, as this can ignore the moral responsibilities of medical professionals. However, at the same time, we regularly prescribe medications, such as acetaminophen, without fully understanding the mechanism [22]. It is argued that, without pursuing the ‘explainability’ of AI tools, better outcomes for patients cannot be provided [23–25].

In addition, many claim that carrying out the decision-making process by a ‘black box’ system carries inherent dangers [26–28]. However, the importance of these dangers varies based on the ethical burden of the decisions that depend on it, thus suggesting that not knowing what a model is based on does not necessarily mean it should be seen as a danger [29]. For example, in the prediction of postoperative mortality, model explainability might be of more value than the prediction of postoperative delayed gastric emptying after distal gastrectomy. As Aristotle stated over two millennia ago, “the ability to verify results by empirical means are more important than to explain the etiology of these results.” This is particularly important in a field in which knowledge of causality is often incomplete, as with postoperative sequalae [21].

Proving that a complication can be predicted while having the ability to reproduce these results might be more important than how this prediction is made. Therefore, a more complex and less explanatory model than a transparent, but simple model might be acceptable for predicting complications [30]. However, to maximize the model interpretability, one could use either individual conditional expectations (ICEs), local interpretable model-agnostic explanations (LIMEs), or Shapley additive explanations (SHAPs) [31]. These techniques aim to increase the comprehensibility of the rationale behind the model’s prediction by visualizing the contributing impact of different variables. This offers the possibility to approach the mechanisms within black-box systems in a way that empowers the clinician to trust the results produced by these models.

### Training, validating, and testing

The generalizability of an ML model depends on the extent and quality of its training, validating, and testing. A very complex model for predicting postoperative mortality after pancreatic surgery might perform nearly perfectly during training but might not be able to properly predict the risk prospectively. This phenomenon is called overfitting, and it occurs when a model is incapable of capturing the relationship between the input variables and the target output values.

One way to estimate the extent of overfitting is through repeated cross-validation or preferentially via bootstrap resampling. This is where the entire modelling process is repeated in each bootstrap replicate. Quantifying the degree of overoptimism in the model’s performance also enables the observation of bias-corrected model performance estimates [20, 32]. Bootstrap resampling with preferentially 200 to 1000 bootstrap replications can provide stable and accurate overoptimism-corrected performance estimates, which has made it the gold standard for internal validation [33, 34]. In contrast, when there is a high error rate in both the training and testing data due to high bias and low variance, the model may be underfit. The balance between underfitting and overfitting is called the bias-variance trade-off [35]. This trade-off shows that when the complexity of a model increases, variance also increases, and bias falls.

### Infrastructure and transparency

Adapting facility infrastructure to enable safe, real-time interaction between the patient file and ML models is both time-consuming and expensive [36]. The implementation of working models in other facilities can be equally difficult since models are often facility-specific [10, 12, 36, 37]. It is therefore of paramount importance that ML models be trained and validated within multiple healthcare facilities with adequate sample sizes to ensure generalizability as well as to prevent substantial harm to the patient [38–40]. In addition to such efforts, the use of a uniform classification system, or international consensus, is a prerequisite for ensuring clinical applicability and generalizability [37].

While abundant literature exists on the methodology and reporting quality of models using conventional statistics (i.e. logistic regression), there is increasing concern about the transparency of studies using ML models [17, 41]. Suboptimal transparency in model development makes ML models hard to interpret, which thus impedes their implementation. This is regarded as the main reason for the limited application of ML models in daily clinical practice [42, 43]. In addition to the Transparent Reporting of a multivariable prediction model for Individual Prognosis or Diagnosis statement (TRIPOD), a protocol for ML-specific prediction was recently published to optimize the transparency of prediction models [17, 44]. Adherence to this statement will improve the interpretability, reproducibility, risk of bias assessment, and ultimately its applicability in clinical practice [45]. The completeness of this checklist is generally poor, as only 38.7% of the 152 articles published using ML models for prediction adhered to the TRIPOD items [16]. Model specification alongside model performance, which are both essential in transparency reports, were also rarely reported [16].

### Data and interpretability

The large effect of the data quality on the final results is an often-mentioned pitfall in AI implementation and is called the Garbage-In-Garbage-Out principle [46]. Inaccurate data directly lead to unreliable results. The World Health Organization stated that proper data quality is multidimensional and should be accurate, available, complete, and valid [46–48]. This quality should always be accurate for ML purposes. The type of data used to feed the model depends on the moment a prediction must be made. Xue et al. [49] evaluated the utility of pre- and intraoperative data for predicting postoperative complications. They concluded that having a combination of pre- and intraoperative data resulted in slightly better performance than an analysis with only one of the two types.

Furthermore, a combination of structured and unstructured data is advisable. With unstructured data in particular, such as data from electronic health records and computed tomography or magnetic resonance imaging, ML models show their superiority [50]. It is therefore advisable to use a combination of both data types when possible to help formulate a risk score based on as many contributing static and modifiable risk factors as possible, allowing for early intervention. When risk scores are created, it is essential that several experts in the field of surgery, nursery, or data science discuss the favorable cut-off values together. The previously discussed ICEs, LIMEs, and SHAPs can also contribute to the interpretability of the model’s output.

### Acceptance and ethical considerations

AI has great potential utility for healthcare professionals in supporting or augmenting clinical decision-making. Multiple studies suggest that these models will play a critical role in future surgical decision-making [51]. However, even when a model has been tested and validated correctly, its degree of acceptance by clinicians and patients can greatly affect its implementation.

Patients correlate AI with science fiction, drawing a fear of machines and computers taking over the making of decisions affecting human beings. The majority of patients thus prefer to receive a healthcare provider’s supervision over AI [52]. The fear of clinicians being replaced by AI leads to mistrust of these models by patients. Similarly, in a study on the acceptance of AI amongst clinicians, only 25% of radiologists had confidence in the results of diagnoses made by AI algorithms [53].

Therefore, medical tools using AI should be used in an assistive manner as opposed to being ultimately responsible for the main decision-making [54]. The ‘doctor in the loop’ is responsible and this responsibility is classified into the following: accountability, liability, and culpability [54, 55]. To tackle this problem it is important to have proper patient education to reassure that the AI systems are not replacing the decision-making of the professional and are merely acting in a complementary manner to them.

Before the predictions of ML models can be used in daily clinical practice successfully, healthcare providers need to achieve trust in these techniques. Surgeons deciding to restore colonic continuity after resection of colon cancer based on an intraoperative ML model predicting a high risk of anastomotic leakage will want to rely on algorithms that have been validated at their own institute. A prospective simulation study in which the predictive performance of a model is tested in addition to the regular local care without affecting its course would enable the correct calibration of the ML model. A calibration curve shows whether or not the predicted chance matches the actual population-based chance of developing a postoperative complication. This approach would allow surgeons to attain trust in the effectiveness and predictive performance of ML models and use them in their clinical practice [56, 57].

The introduction of ML models has led to an unprecedented amount of ethical issues, and guidelines regarding these ethical considerations are still sparse [58]. Currently available frameworks for governance were discussed in a recently published review of the literature [59]. This study included 21 guidelines for gold-standard societal values, such as sustainability, freedom, and fairness. Although these guidelines appeared to be insufficient when analyzed separately, it was stated that the ideal rules for ethical considerations should harmonize interests, offering benefit to clinicians, patients, and hospitals [59]. A governance model for the application of ML models in healthcare based on the abovementioned concept was developed recently [60]. In our opinion, it is of utmost importance to adhere to such governance models to ensure acceptance as well as ethical and legal appropriateness.

Correct prediction of postoperative complications using ML has the potential to dramatically improve the outcome of everyday clinical surgical care. However, their implications for patients should be considered before implementing prediction models in clinical practice. For example, predicting anastomotic leakage after colorectal surgery may lead to more stomas or earlier discharge when leakage is not expected to occur. This could also lead to collateral over- and under-treatment. This change in the paradigm of clinical practice must be accepted by all healthcare providers to ensure full benefit from these techniques. Therefore, it is advisable to obtain solid prospective validation from external sources at different centers with adequate sample sizes, all while adhering to the transparency and ethical guidelines to overcome potential distrust concerning ML among clinicians and patients.
